# The Mitochondrial Outer Membrane Protein Tom70-Mediator in Protein Traffic, Membrane Contact Sites and Innate Immunity

**DOI:** 10.3390/ijms21197262

**Published:** 2020-10-01

**Authors:** Sebastian Kreimendahl, Joachim Rassow

**Affiliations:** Institute for Biochemistry and Pathobiochemistry, Ruhr-University Bochum, 44801 Bochum, Germany; sebastian.kreimendahl@rub.de

**Keywords:** Tom70, mitochondria, mitochondrial protein import, mitochondrial carrier family, SLC25, membrane contact sites, SARS-CoV-2, MAVS, Lam6, Ltc1, IP3R3

## Abstract

Tom70 is a versatile adaptor protein of 70 kDa anchored in the outer membrane of mitochondria in metazoa, fungi and amoeba. The tertiary structure was resolved for the Tom70 of yeast, showing 26 α-helices, most of them participating in the formation of 11 tetratricopeptide repeat (TPR) motifs. Tom70 serves as a docking site for cytosolic chaperone proteins and co-chaperones and is thereby involved in the uptake of newly synthesized chaperone-bound proteins in mitochondrial biogenesis. In yeast, Tom70 additionally mediates ER-mitochondria contacts via binding to sterol transporter Lam6/Ltc1. In mammalian cells, TOM70 promotes endoplasmic reticulum (ER) to mitochondria Ca^2+^ transfer by association with the inositol-1,4,5-triphosphate receptor type 3 (IP3R3). TOM70 is specifically targeted by the Bcl-2-related protein MCL-1 that acts as an anti-apoptotic protein in macrophages infected by intracellular pathogens, but also in many cancer cells. By participating in the recruitment of PINK1 and the E3 ubiquitin ligase Parkin, TOM70 can be implicated in the development of Parkinson’s disease. TOM70 acts as receptor of the mitochondrial antiviral-signaling protein (MAVS) and thereby participates in the corresponding system of innate immunity against viral infections. The protein encoded by Orf9b in the genome of SARS-CoV-2 binds to TOM70, probably compromising the synthesis of type I interferons.

## 1. Introduction

Mitochondria have fascinated researchers across all disciplines of life science for over a century [1]. These eukaryotic organelles most likely originate from an aerobic bacterium, related to modern α-proteobacteria, that was incorporated by the progenitor of the eukaryotic cell. In the development of the endosymbiotic relationship, the bacterium retained its ring-shaped chromosome but lost most of its genes. In all eukaryotes, approximately 99% of the mitochondrial proteins are encoded by the nuclear DNA and synthesized by cytosolic ribosomes [2,3,4]. The efficient selection and uptake of newly synthesized proteins is mediated by an elaborate machinery in the mitochondrial outer and inner membranes in cooperation with several soluble factors. The decisive entry gate is formed by a complex of outer membrane proteins named TOM complex (translocase of the mitochondrial outer membrane [5]). A prominent component of the TOM complex, albeit only loosely associated, is Tom70.

The mitochondrial proteome comprises ~1000 (yeast)-1500 (human) different proteins. In the yeast *Saccharomyces cerevisiae*, 74 integral and 21 peripheral outer mitochondrial membrane proteins were identified by a sophisticated proteomic approach [6,7,8]. Tom70 is an abundant protein of the mitochondrial outer membrane, and in *S. cerevisiae*, the corresponding gene (gene: YNL121C; UniProtKB-P07213; synonym: MAS70) was identified already in 1983 [9,10,11]. The characterization of Tom70 as an import receptor for a subset of mitochondrial proteins followed several years later: Studies on the import of proteins into the mitochondria of the filamentous fungus *Neurospora crassa* revealed a role of Tom70 (gene: NCU04245; UniProtKB–P23231; synonym: MOM72) in binding of newly synthesized metabolite carrier proteins such as the ADP/ATP carrier of the mitochondrial inner membrane [12], and this function was confirmed for the Tom70 of yeast [13,14]. In parallel, the outer membrane protein Tom20 was identified as a receptor for precursor proteins targeted by an amino terminal presequence and Tom40 was characterized as the central pore-forming component of the TOM complex. Since then, the system of the two import receptors Tom70 and Tom20 in cooperation with the channel-forming Tom40 was regarded as the central structure the TOM complex. Subsequent studies showed that the TOM complex contains several additional components and cooperates with independent complexes of other outer membrane proteins, but they essentially confirmed the basic scheme of distinct receptor proteins, one of them being Tom70, cooperating with a general import pore [3,4] (see below, Figure 5). Although Tom70 functionally cooperates with the core components of the TOM complex to facilitate import of mitochondrial proteins, it is not permanently associated with Tom40 [13,15,16,17,18,19,20]. The molecular structure of the TOM complex was recently resolved by high resolution Cryo-EM [21,22,23]. The studies confirmed the tight and stable association of Tom40 with several additional Tom proteins, but Tom70 was not included. Tom70 is thus an outer membrane protein that associates with Tom40 only partially and in a reversible manner.

**Figure 1:**
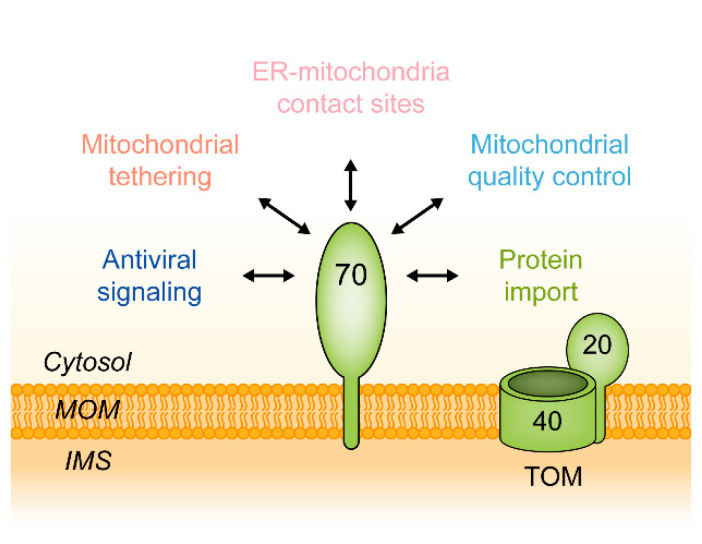
Cellular functions of the mitochondrial outer membrane protein Tom70. IMS, intermembrane space; MOM, mitochondrial outer membrane; TOM, translocase of the mitochondrial outer membrane, containing the channel-forming protein Tom40 and the import receptor Tom20.

But why is it useful to review the current state of research on a protein that was characterized 30 years ago, if the basic scheme of its function seems to be retained? In fact, in recent years, a wealth of new data has emerged, showing that Tom70 has additional functions, some of them being entirely independent of the biogenesis of mitochondrial proteins: (1.) In 2006, the first high resolution crystal structure of a Tom70 was published [24]. (2.) Already in 2003, data had revealed a defined binding site within Tom70 for cytosolic Hsp70 [25]. (3.) A series of new studies on tetratricopeptide (TPR) domain-containing proteins of the endoplasmic reticulum and of chloroplasts revealed striking similarities to Tom70 both in the tertiary structure and also in the function as a receptor for heat shock proteins. (4.) In 2011, the activity of Tom70 in binding of preproteins was shown to be regulated by reversible phosphorylation of a distinct serine residue [26]. (5.) The advent of whole genome sequencing allowed a first insight into the evolution of Tom70 in different eukaryotic lineages. (6.) Tom70 was found to participate in direct interactions between mitochondria and partner proteins of the endoplasmic reticulum. (7.) TOM70 interacts with the mitochondrial antiviral-signaling protein (MAVS), a component in a system of antiviral immunity, and some data indicate that human TOM70 is a target of a protein encoded by the genome of the SARS-CoV-2 (the severe acute respiratory syndrome coronavirus 2) [27,28]. (8.) Eventually, new investigations confirm that Tom70 facilitates the mitochondrial import of many different proteins, but they also indicate that the specific selection of proteins by the TOM complex is independent of Tom70 [29]. Together, the new observations demonstrate that Tom70 is a versatile mediator in protein traffic, membrane contact sites and signaling (Figure 1).

## 2. Molecular Structure of Tom70

### 2.1. The Structure of Monomeric Tom70

Yeast Tom70 is a 70.1 kDa protein of 617 amino acids anchored in the mitochondrial outer membrane. The membrane anchor is located in the extreme N-terminus, which harbors a hydrophobic segment (between amino acids 9 and 38) whereas the hydrophilic receptor domain is exposed in the cytosol [11,13]. The insertion of newly synthesized Tom70 into the outer membrane is mediated by the MIM complex and proceeds independently of both Tom20 and Tom70 of the TOM complex [30,31].

**Figure 2:**
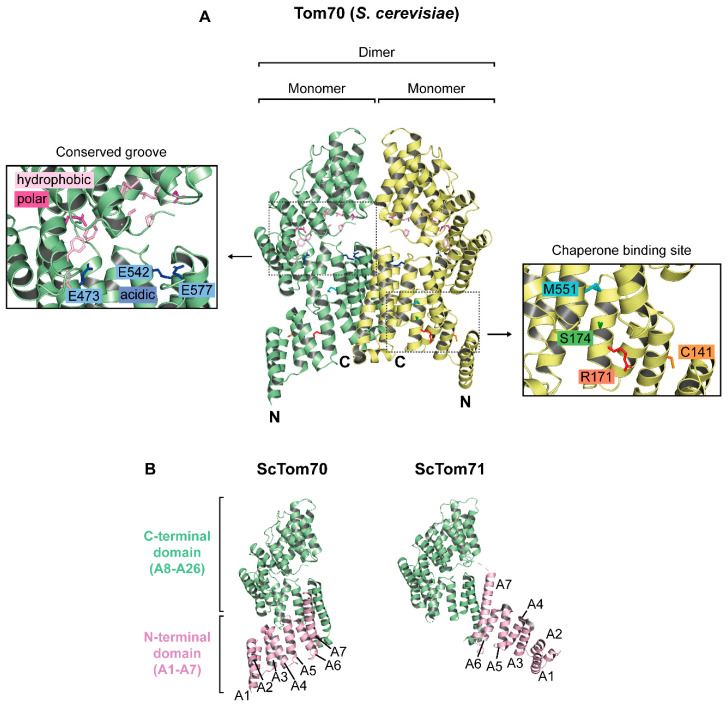
Overview of the structural features of *S. cerevisiae* Tom70. (**A**): Crystal structure of dimeric Tom70. Structural data were obtained from Wu & Sha (2006). Each Tom70 monomer contains 617 amino acids and has a molecular mass of approximately 70 kDa. The secondary structure of Tom70 is characterized by 26 α-helices (A1–A26) that form 11 tetratricopeptide repeat (TPR) motifs and create its supra-helical tertiary structure. The N-terminal domain (A1–A7) of Tom70 contains a chaperone binding site formed by a clamp-type TPR domain. The binding site is defined by an arginine at position 171 and a cysteine at position 141 [25,29]. The TPR clamp contains a serine at position 174 which can be phosphorylated by protein kinase A (PKA) in response to metabolic changes [26]. Within its C-terminus, Tom70 contains a highly conserved groove which displays mainly hydrophobic and a few polar residues at its top and three conserved glutamates at its bottom. The opposite site of the TPR clamp domain contains a methionine at position 551 which might be important for presequence recognition by Tom70 [40]. (**B**): Configurations of the N-terminal domains (A1–A7) of *S. cerevisiae* Tom70 [24] and Tom71 [37]. The different orientations of the N-terminal domain relative to the C-terminal domain are suggested to indicate that both proteins may adopt different conformational states.

A crystal structure of yeast Tom70 at a resolution of 3.0 Å was provided in a landmark publication by Wu & Sha (2006). The crystal structure revealed that Tom70 is essentially a bundle of 26 α-helices (A1–A26), of which the majority is involved in the formation of 11 tetratricopeptide repeat (TPR) motifs (Figure 2A). A Tom70 monomer forms a supra-helical structure with two distinct parts: An N-terminal domain (helices A1–A7) and a C-terminal domain (helices A8–A26) (Figure 2B). The Tom70 superhelix has a length of approximately 100 Å and a radius of 50 Å. The N-terminal and the C-terminal domain are connected to each other in a “head to head” orientation, where the C-terminal ends of both domains face each other with antiparallel helices A7 and A25 [24].

A defined chaperone binding site is contained in the N-terminal domain of Tom70: The helices A1–A6 (TPR motifs 1–3) form a clamp-type TPR domain that serves as a chaperone acceptor for Hsp70 in yeast and for Hsp70 and Hsp90 in mammals [24,25,32,33].

In parallel to Tom70, the genome of *S. cerevisiae* encodes a functional paralog, Tom71, which is expressed at low levels [34,35,36]. In comparison to Tom70, Tom71 was found to display a strikingly divergent arrangement of its C- and N-terminal domains. The differences seem to refer to alternative conformational states of both Tom70 and Tom71, including an open and a closed conformation (Figure 2B), and this flexibility was suggested to determine the accessibility of a binding site for protein recognition [37,38,39,40].

### 2.2. Putative Preprotein Binding Sites

It is traditionally assumed that Tom70 not only binds, but also selects a subset of mitochondrial preproteins by direct interactions with structures that are specific for these proteins. The essential receptor sites within the structure of Tom70 that serve this purpose are still unclear, although possible binding sites were characterized in several studies:

An early study reported a stably folded 25 kDa core domain of Tom70 (amino acids 247–460, hence outside the chaperone binding site), that was able to bind to chemically synthesized internal segments of substrate proteins in vitro with a specificity comparable to the full length receptor [41]. The assays of this comprehensive study revealed a distinct pattern of affinities, but the relevance of these affinities for chaperone-mediated binding of preproteins to Tom70 in vivo was not investigated.

Analyzing the crystal structure of yeast Tom70, Wu & Sha (2006) identified a highly conserved groove located in the center of its C-terminal domain (containing TRP motifs 4–11) [24]. While the distal side of the binding groove is mainly made up of conserved hydrophobic and polar residues (hydrophobic: Pro252, Phe260, Phe341, Leu342, Met406, Phe408, Ile409, Phe432, Phe470, Ile474; polar: Asp375, Gln436, Gln405), the proximal side contains three conserved residues that are negatively charged (Glu473, Glu542, Glu577). The authors suggested this groove to represent the major binding site for preproteins, with the conserved hydrophobic parts acting as docking site for the hydrophobic substrates of Tom70 [24]. However, this function has not yet been confirmed by experimental evidence.

In a study on the role of Tom70 in the mitochondrial import of carrier proteins, Tom70 variants were included with the highly conserved glutamates in the proposed binding groove exchanged against alanine. The experiments did not show any indication of an involvement of this structure in carrier protein binding or selection [29]. An independent study found that presequence peptides can be photo-crosslinked to residues at least in close proximity to the postulated binding groove [40]. A variant of Tom70 in which the Met551 was replaced by an arginine showed reduced affinity to Mdl1 (a presequence-containing precursor) but did not impair the import of carrier proteins. The results of this study are in agreement with a possible role of Tom70 in direct interactions with presequences of precursor proteins, but the role of the postulated binding groove in these interactions is still unclear.

In retrospect, the data on binding sites that may determine a specificity of Tom70 in the selection of subsets of mitochondrial preproteins do not provide a clear picture. The only site of Tom70 that unambiguously serves as a docking site for preproteins is thus the site within the N-terminal part of Tom70 that is able to bind Hsp70 and Hsp70-bound preproteins [25,29]. The function of Tom70 in mitochondrial protein import is regulated by reversible phosphorylation of a serine which is located in the center of this chaperone binding site [26]. Remarkably, the accessibility of this site seems to be directly affected by the transition between the closed and the open conformation, i.e., by the movement of helices A1–A6 out of the supra-helical structure of the protein [37].

Figure 2 gives an overview over the structural features of yeast Tom70, including the relevant amino acid residues in the segment suggested as putative binding groove by Wu & Sha (2006) and in the chaperone binding site identified by Young et al., (2003).

### 2.3. The Oligomeric State of Tom70

Biophysical studies indicate that recombinant yeast Tom70 is a monomer in aqueous solution [39,42]. However, in the outer membrane of yeast mitochondria, Tom70 is mainly organized in functional homo-oligomers. Radio-labeled AAC translocation intermediates that were chemically cross-linked at the surface of yeast mitochondria were mainly found in association with Tom70 homodimers [43,44]. Complexes of up to six Tom70 molecules were resolved in a study using native gel electrophoresis (BN-PAGE), suggesting that in yeast each of the three modules of a carrier protein can bind to a dimer of Tom70 [45]. The same study was also able to identify Tom70 dimers even in the absence of a preprotein.

The elements of Tom70 that mediate its oligomerization in the membrane are unclear. The crystal structure of yeast Tom70 shows dimers that are stabilized by several hydrophobic residues within helices A6 and A7 of the N-terminus of one monomer with hydrophobic residues of helices A25 and A26 of the C-terminal domain of the opposing monomer [24]. However, also the N-terminal membrane spanning segment of yeast Tom70 seems to have a tendency to dimerize [46,47]. The structures that determine the association of Tom70 monomers with each other in intact mitochondria have not been elucidated. Isolated human TOM70 in solution seems to exist in an equilibrium between the monomeric and dimeric form and might also function as a monomer in its membrane environment [48]. This raises the question, if the functional oligomeric state of Tom70 may differ between yeast and mammals. Interestingly, in contrast to yeast Tom70, its paralog Tom71 crystallizes as a monomer in the postulated open conformation [37]. Unfortunately, a crystal structure of mammalian TOM70 is not yet available.

## 3. Evolution of Tom70

### 3.1. Tom70 and Its Homologs

Although many components of the mitochondrial import machinery evolved from proteins of their bacterial ancestors [49] and bacteria and archaea contain many TPR proteins, no prokaryotic homolog of Tom70 has been identified so far [50]. Tom70 was identified only in certain eukaryotic lineages, especially in animals and fungi, but not in plants [51].

**Figure 3:**
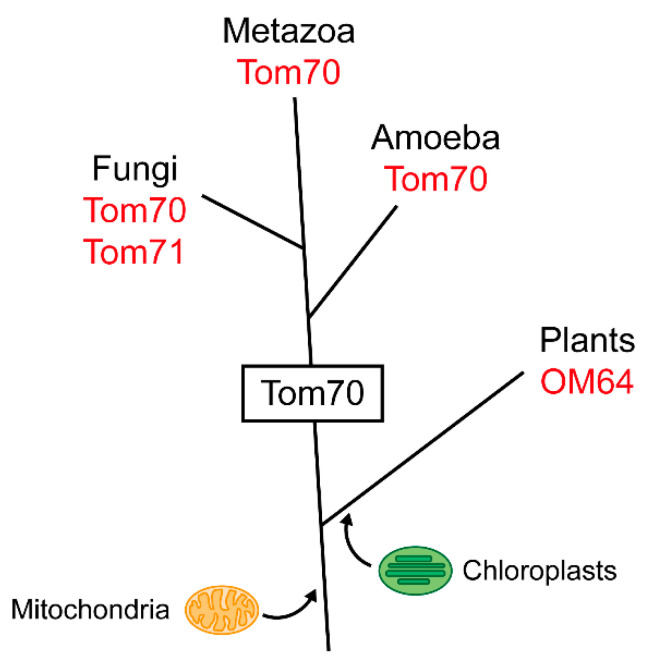
Tom70 and its functional analogs in different eukaryotic lineages. The mitochondrial outer membrane protein Tom70 is found in the eukaryotic kingdoms of metazoa, fungi and amoeba. Mitochondria of plants are lacking a Tom70, but they contain the outer membrane protein OM64, an unrelated TPR protein that serves similar functions.

Tom70 obviously evolved as an additional protein of the mitochondrial outer membrane at a distinct time during the evolution of the eukaryotes (Figure 3). Animals are closer related to fungi than to plants, which explains the striking similarity between the extensively studied mitochondrial protein machinery of *N. crassa* and *S. cerevisiae* and the import machinery of human mitochondria [52,53]. Mammalian Tom70 proteins closely resemble the homologous proteins of fungi [54,55,56,57]. However, human TOM70 (GenBank Accession Number: AB018262; UniProtKB-O94826) has additional functions, possibly exclusive to mammals [58]. The yeast *S. cerevisiae* possesses a functional paralog of Tom70, named Tom71, which is likely a result of a whole genome duplication and has no counterpart in mammals [34,35,59].

Protists are a heterogeneous group of unicellular eukaryotic organisms and their heterogeneity is reflected in their content of Tom70 homologs. Amoeba have traditionally been classified as protists and are now recognized as closely related to fungi and animals [53,60]. Consistently, Tom70 was identified in *Acanthamoeba castellanii* and *Dicytostelium discoideum* [61,62]. In contrast, Tom70 is virtually absent in the phylogenetically separate supergroup Excavata which includes *Trichomonas vaginalis* and *Trypanosoma brucei* [63,64]. Within the eukaryotes, the stramenopiles comprise a distinct group that mainly contains algae but is phylogenetically difficult to classify. Surprisingly, a study found a Tom70 homolog in mitochondria-like organelles of the anerobic parasitic stramenopile *Blastocystis sp*. [65]. This led the authors to reevaluate the evolutionary distance between stramenopiles and other eukaryotes, emphasizing the relation to the Tom70-containing animals and fungi.

Plant mitochondria import proteins independently of a Tom70 homolog, suggesting that a Tom70 had not yet been developed when primordial cyanobacteria were incorporated by eukaryotic ancestors, thereby initiating the evolution of plastids [66,67]. The lack of Tom70 in plant mitochondria indicates that Tom70 was a new protein that emerged after the divergence of the plant lineage from a common eukaryotic ancestor, but prior to the separation of animals, fungi and amoeba (Figure 3).

Interestingly, in many lineages lacking a Tom70 homolog, alternative TPR receptors evolved that seem to exert a similar function at the mitochondrial outer membrane. For instance, in *Trypanosoma brucei* the mitochondrial outer membrane protein ATOM69 is a TPR receptor protein that cooperates with a pore-forming β-barrel protein that has no sequence homology to Tom40 but a similar function [63]. In the plant kingdom, functional analogs of Tom70 have evolved independently that act within chaperone-guided protein targeting systems [67,68].

Given that plants contain fully intact mitochondria with several homologs of the animal or fungi protein import machinery, including Tom20 and Tom40, it is remarkable that Tom70 arose rather late during the eukaryotic evolution [69]. The import of proteins into mitochondria of plant cells or trypanosomes works well without any Tom70 homolog, with other TPR proteins acting as alternative protein import receptors. However, it is tempting to speculate that Tom70 may have provided unique opportunities in the evolution of further cellular functions. Perhaps Tom70 was particularly suitable to act as a flexible adaptor protein within complex systems of intracellular interactions, thereby contributing to the extraordinarily productive era of the evolution known as the Cambrian explosion and to the development of the metazoa into a diverse group of eminently complex organisms.

### 3.2. The TPR Domain of Tom70 and Its Functional Analogs in Other Membranes

Almost every protein import complex of organellar membrane systems contains a tetratricopeptide repeat (TPR) protein to enhance the efficiency of protein translocation [67]. Therefore, it is worthwhile to take a closer look at the functions and structural hallmarks of the TPR domains found in Tom70 and its functional analogs in other membranes.

A TPR domain consist of up to 20 tetratricopeptide repeats, each comprising 34 amino acids which adopt a helix-turn-helix motif [70,71,72,73]. The two α-helices of a single TPR motif display a packing angle of roughly 24° [74]. Therefore, stacked TPR motifs induce a right-handed, super-helical conformation of a protein, which is also the predominant structure of Tom70. TPR motifs are mediators of protein-protein interactions and are involved in a plethora of cellular functions that go beyond the transport of proteins [75]. The consensus sequence of TPR motifs is mainly made up of large and small hydrophobic amino acids and is highly conserved only at a few positions that ensure the structure of the domain. However, the tertiary structures of different TPRs resemble each other and their conserved configuration can be regarded as a functional neutral scaffold, whose binding specificity is defined by incorporation of functionally specific amino acids [76].

The specialized TPR domain involved in recognition of chaperones of the Hsp70 family is often referred to as “carboxylate clamp” because it forms a binding site for short segments of polypeptide chains that contain a series of carboxyl groups. Common ligands of the carboxylate clamp-type TPR domain are short peptides, like the EEVD motif found in the C-terminus of Hsp70/Hsp90 proteins [72]. Examples of clamp-type TPR domains are found in co-chaperones Hip (mammals), Hop (mammals and yeast) or Tom70 (TPR domains 1–3) [25,32].

**Figure 4:**
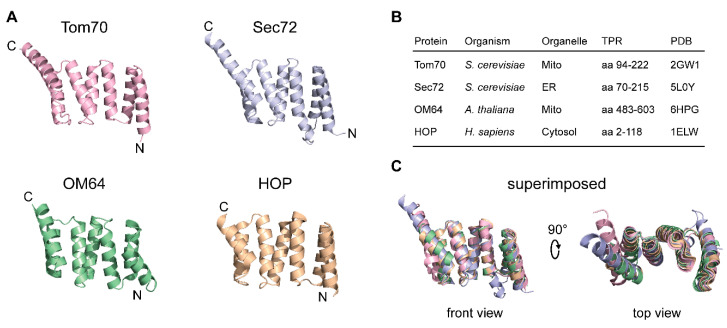
Comparison of clamp-type TPR domains of *S. cerevisiae* Tom70 and functional analogs in other membrane systems. (**A**): Sec72 mediates the recognition of Ssa1 at the ER membrane in yeast. OM64 mediates protein recognition and import across the outer chloroplast membrane in analogy to Tom70. Mammalian co-chaperone HOP is a classic example for a clamp-type TPR protein and acts as an adapter protein to facilitate interaction of Hsp70 and Hsp90. (**B**): Polypeptide segments depicted in (**A**). (**C**): Superimposed model of the TPR domains of Tom70, Sec72, OM64 and HOP. The images were created with the PyMOL Molecular Graphics System, Version 2.0 (Schrödinger) using the structural data annotated in (**B**). TPR, tetratricopeptide repeat; ER, endoplasmic reticulum.

TPR domain receptors are not an exclusive feature of the mitochondrial outer membrane but are involved in protein targeting to different organelles, often by aiding the recognition of preproteins via binding of associated chaperones like Hsp70. Although Tom70 analogs in other organelles arose independently, their clamp-type TPR domains display a striking resemblance (Figure 4).

Sec72 is part of the protein complex that recognizes proteins for post-translational import into the endoplasmic reticulum (ER) in *S. cerevisiae* [77]. Besides Sec72, the complex comprises the import channel Sec61 and the associated proteins Sec62, Sec63, Sec71. Sec72 accepts ER-proteins bound to Ssa1 (yeast Hsp70) and facilitates their translocation through the Sec61 channel into the ER lumen. Thus, Sec72 exerts a function comparable to Tom70 on the mitochondrial surface.

In plants, the uptake of proteins into mitochondria is independent of any Tom70 homolog [66,67,69]. However, plants possess a unique TPR receptor in their mitochondrial outer membrane, OM64, which is involved in protein import by interactions with preproteins bound to Hsp70 and Hsp90 and thus substitutes for the lack of Tom70 [78,79]. Interestingly, the OM64 function is also regulated by a phosphorylation site within its TPR domain, similar to Tom70 [80]. OM64 itself is a homolog of Toc64 (translocase of the outer chloroplast membrane), the TPR receptor of the import complex of chloroplasts [78,81]. Toc64 works in analogy to Tom70 on the chloroplast surface of plant cells: it contains a C-terminal carboxylate clamp-type TPR domain that recognizes preproteins by interaction with Hsp70 and Hsp90 [68,82,83,84]. Similar to Tom70, Toc64 is not essential for the import of preproteins [85].

In contrast to the aforementioned TPR receptors of cellular membranes, the cytosolic TPR receptor Pex5 does not recognize negatively charged EEVD motifs of chaperones, but the peroxisomal targeting signal 1 (PTS1) which is defined by a C-terminal peptide with the consensus sequence Ser-Lys-Leu-COO^−^ [86,87,88].

In *S. cerevisiae*, the majority of TPR proteins contains 3–8 TPR motifs, with an average of 5 TPRs [76]. In comparison, Tom70 contains 11 TPRs, and thus significantly more than most other TPR proteins, thereby facilitating a cooperation of Tom70 with different interaction partners.

## 4. Functions of Tom70 in the Biogenesis of Mitochondrial Proteins

### 4.1. Tom70 and Chaperone Proteins

Tom70 is regarded as the major import receptor for newly synthesized metabolite carriers of the mitochondrial inner membrane [4,12,13,14,45,89,90,91]. The mitochondrial carrier family (MCF; in mammals: solute carrier family 25, SLC25) is a large group of transporters that comprises 35 members in *S. cerevisiae* and 53 members in *H. sapiens* [92]. Most studies on these proteins were carried out using the ADP/ATP carrier (AAC) as a model protein. However, other proteins have been described as additional Tom70 substrates, namely the β-barrel proteins of the mitochondrial outer membrane, single- and multi-membrane-spanning (polytopic) proteins and some non-canonical carriers [93,94,95,96,97,98]. Hence, the substrates of Tom70 are obviously diverse, but most of these proteins contain highly hydrophobic domains and thus have the tendency to aggregate outside their target membrane. This is also a feature of the inner membrane metabolite carriers which typically contain 6 transmembrane domains [99,100].

Upon synthesis in the cytosol, the hydrophobic proteins bind to chaperones, which prevent their aggregation and keep the proteins in an import-competent state. The complex of the chaperone and the preprotein then binds to the chaperone-binding site of Tom70 [101]. Whereas in yeast primarily Hsp70 plays a role in mitochondrial targeting, in mammals Hsp90 is participating in this process as well [25].

Binding of Hsp70 and Hsp90 to Tom70/TOM70 is mediated by a negatively charged EEVD motif in the C-terminus of the chaperones, which interacts with positively charged amino acids in the chaperone binding site formed by the N-terminal clamp-type TPR domain (TPR 1–3) comprised of the helices A1–A6 of the receptor [24,25,102]. A study combining chemical crosslinking of Hsp90 and TOM70 with a subsequent mass spectrometry analysis found an additional segment of Hsp90 (amino acids 654–660) to be cross-linked to a second interaction site of TOM70, independently of helices A1–A6 [103]. This proposed second interaction site involves the lysins 199, 203, 233, and 319 of TOM70 and suggests a participation of helix A7 in Hsp90 binding. This model of two interaction sites between Hsp90 and TOM70 is supported by biophysical experiments indicating that Hsp90 binds to TOM70 in a dimeric fashion [104]. In yeast Tom70, an arginine at position 171 was found to be essential for the interaction with the Hsp70 EEVD motif [25]. In close proximity to Arg171 a serine residue at position 174 can be phosphorylated by protein kinase A (PKA), which mitigates binding of Hsp70 and impairs the import of chaperone-bound Tom70 substrates [26]. Blocking of a cysteine at position 141 in the direct vicinity to Arg171 and Ser174 with N-ethylmaleinimide (NEM) completely inhibits Tom70 function during the selection of newly synthesized carriers from the cytosol [29].

While the translocation of proteins over the mitochondrial membranes has been studied for decades, many molecular details of the cytosolic events involved in mitochondrial targeting are still unclear [105]. However, in the past few years several important studies were devoted to the elucidation of these mechanisms and uncovered an important role of co-chaperones in targeting of proteins to mitochondria [106]. In yeast, Djp1 (a member of the Hsp40/DnaJ family) promotes the delivery of hydrophobic preproteins to mitochondria by direct binding to Tom70 [107,108]. Newly synthesized β-barrel-forming proteins of the mitochondrial outer membrane are bound to Hsp70 and subsequently recognized by Tom70, a process in which the co-chaperones Ydj1 and Sis1 (likewise members of the yeast Hsp40/DnaJ family) are involved [109]. In addition to Ydj1 and Sis1, the co-chaperone Sti1 was found to be able bind to the purified cytosolic domain of Tom70 in vitro [110]. In humans, several co-chaperones of the Hsp40-related DJA family (DJA1, DJA2 and DJA4) are components of the chaperone complex that binds to the adenine nucleotide transporter (ANT; the mammalian homolog of the yeast AAC), however, a direct interaction with TOM70 was not tested [111].

### 4.2. Tom70 and Targeting Signals

Because cytosolic chaperones are involved in targeting of proteins to different organelles, additional targeting signals are required to direct the individual proteins to a defined target membrane. In most cases, Tom70 is involved in the uptake of preproteins that are devoid of a positively charged presequence, hence, Tom70 seems to recognize other structural features to select its substrate proteins. But what are these features? In fact, it has been difficult to answer this question even with respect to the mitochondrial carrier family, by far the largest group of proteins that bind to Tom70 [4].

The members of the mitochondrial carrier family (MCF) are composed of three similar modules, that are connected to each other by short hydrophilic loops. Each module is embedded in the target membrane by two transmembrane domains connected by a matrix loop containing a surplus of positively charged amino acid residues. In most cases, the N-terminal part of each matrix loop contains a conserved motif, PX(D/E)XX(K/R), which is known as the carrier signature [112]. Some mammalian carriers possess an N-terminal presequence, however, neither the carrier signature, nor the presequences are essential elements for mitochondrial targeting [113,114,115,116,117]. Based on the observation that even single modules can show specific mitochondrial targeting, it was concluded that the carriers possess internal targeting signals distributed within all three modules, but the decisive nature of these signals remained elusive [4,45,118]. The results of a recent study showed that targeting of carrier proteins to mitochondria is mediated by a bipartite targeting signal, which is composed of a positively charged matrix loop followed by a transmembrane domain [29]. The positively charged residues were found to be essential elements of this scheme. Surprisingly, the recognition of this bipartite targeting signal at the mitochondrial surface is independent of Tom70 and of any other cytosol-exposed receptor protein. In line with this observation, it was found that isolated Tom70 was not able to discern the positive charges that are essential elements of the targeting sequence. Since Tom70 is unable to recognize the bipartite targeting sequences, it appears to act merely as a membrane-bound co-chaperone for the cytosolic Hsp70 that is associated with the substrate proteins. Tom70 essentially seems to facilitate the interaction of the bipartite targeting signal with the pore-forming protein Tom40, which shows a preference for cations and thus represents an attractive target for the positive charges of the matrix loops of the carrier modules [119,120,121]. Interestingly, the recently solved Cryo-EM structures of isolated TOM complexes confirmed that the Tom40 channel contains hydrophobic as well as acidic patches, which supports the idea of the import pore recognizing the bipartite targeting signal of carrier proteins [17,22,23]. After the ATP-dependent release from the chaperone/Tom70 complex and translocation through the import pore, carrier proteins are recognized by the small Tim chaperones of the mitochondrial intermembrane space (IMS) and transferred to the TIM22 complex for subsequent incorporation into the mitochondrial inner membrane [122,123,124,125,126,127,128,129,130].

**Figure 5:**
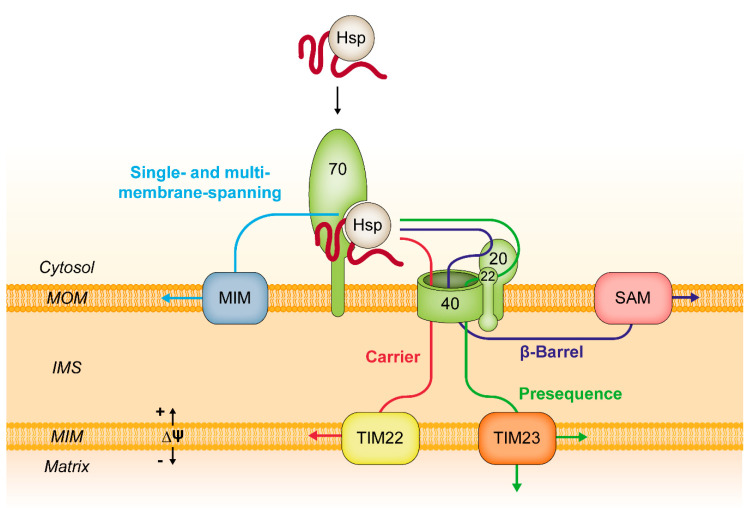
Functions of Tom70 in mitochondrial protein import. Tom70 serves as a docking station for different classes of proteins. Most of these proteins contain hydrophobic domains and are initially associated with chaperone proteins. Tom70 recognizes the chaperone/preprotein complex as a co-chaperone and facilitates the interaction of the preprotein with other components of the mitochondrial import machinery. Carrier proteins are imported through the import pore Tom40 and inserted into the mitochondrial inner membrane by the translocase of the inner mitochondrial membrane (TIM22). Presequence proteins are preferentially recognized by the import receptors Tom20 and Tom22 and pass the mitochondrial outer membrane through Tom40. Import of presequence proteins into the inner membrane or matrix is mediated by the TIM23 complex. Mitochondrial β-barrel proteins interact with the Tom20 receptor, traverse the intermembrane space and are inserted into the outer membrane by the sorting and assembly machinery (SAM). Single- and multi-membrane-spanning proteins of the outer membrane require a cooperation of Tom70 with the mitochondrial import (MIM) complex. Hsp, heat shock protein; IMS, intermembrane space; ∆Ψ, mitochondrial membrane potential.

For the incorporation of α-helical single- or multi-membrane-spanning proteins into the mitochondrial outer membrane, Tom70 does not cooperate with Tom40 but with the MIM complex [96,97,131] (Figure 5). Membrane-spanning α-helical proteins of the outer membrane are lacking the net-positive matrix loop of carrier proteins. The outer membrane protein Ugo1 even displays an overall negatively charged IMS loop [132]. This indicates that Tom70 alone can recruit proteins to the mitochondrial surface, but specific targeting is mediated by separate structures such as Tom40 or the outer membrane MIM complex.

Interestingly, Tom70 also seems to function merely as a co-chaperone in the biogenesis of β-barrel proteins of the mitochondrial outer membrane: The targeting signal of β-barrel proteins, a β-hairpin motif, is recognized by Tom20, whereas the associated chaperones and co-chaperones are recognized by Tom70 [109,133].

Moreover, Tom70 interacts with several mitochondrial presequence-containing proteins [40,51,134,135,136]. Yamamoto et al. [134] studied the mitochondrial import of 114 presequence-containing proteins and found that about 15% were strongly affected by the deletion of the *tom70* gene. In the same study, the authors found that upon synthesis in a cell-free system lacking cytosolic chaperones, the solubility of several of these proteins was increased in the presence of the purified cytosolic domain of Tom70. These observations suggest that at least under experimental conditions, Tom70 can have a chaperone-like function by directly interacting with parts of the precursor proteins [134]. However, it is unclear if Tom70 indeed has a chaperone-like function in vivo. In intact cells the solubility of proteins is usually supported by cytosolic chaperones [106] and Tom70 may preferentially act as a docking station for chaperone-associated preproteins [25]. In fact, interactions of different presequence-targeted proteins with Tom70 may depend on alternative mechanisms: A more recent study demonstrated that the recognition of preproteins by Tom70 can be promoted by mitochondrial presequence-like segments within the mature protein, referred to as internal matrix-targeting signals (iMTS) [137,138]. The iMTS seem to bind to distinct sites of Tom70 independently of associated chaperone proteins.

Taken together, these new studies suggest that Tom70 facilitates binding of hydrophobic substrates to the mitochondrial surface mainly by the recognition of the associated chaperones and co-chaperones rather than the preprotein itself. The association of the chaperone is probably unstable and dependent on the local availability of ATP. This model is in agreement with several studies that found that Tom70 is not an essential component for preprotein import into mitochondria but rather increases its efficiency [89,139,140]. In the biogenesis of mitochondrial proteins, Tom70 seems to act as a relatively unspecific initial docking station for the preprotein/chaperone complex, presumably to raise the local concentration of proteins destined for mitochondria, without itself conveying any tool for selecting preproteins from the cytosol. The selection of mitochondrial proteins by recognition of their targeting signals seems to be a downstream event, mediated by other proteins of the mitochondrial outer membrane (Figure 5).

### 4.3. Tom70 and Co-Translational Protein Import

Prior to any detailed studies on mitochondrial protein import it was already known that cytosolic ribosomes are attached to the mitochondrial outer membrane and it was confirmed that these ribosomes are able to synthesize mitochondrial proteins [141,142]. However, further investigations indicated that most mitochondrial proteins that are synthesized in the cytosol are imported post-translationally [143,144]. It is therefore assumed that the majority of mitochondrial proteins is imported post-translationally, albeit the relative contribution of co- vs. post-translational import has so far not been determined [105,145,146,147]. More recently, studies were conducted to investigate the structures that anchor the ribosomes and mRNAs to the mitochondrial surface. There is now some evidence that Tom70 might play a role in the localized translation of proteins, as the amounts of mitochondria-associated mRNAs and ribosomes are reduced in yeast and in mammalian cells lacking Tom70 [148,149]. However, the means by which Tom70 participates in the recruitment of the translating ribosomes are unclear. Remarkably, also a deletion of the gene encoding Ssa1 (yeast Hsp70) leads to a decrease in mitochondria-localized mRNAs, suggesting that the Tom70/Hsp70 interaction may be relevant in this process [150].

### 4.4. Mitochondrial Protein Import in Cooperation with the Endoplasmic Reticulum

Under certain conditions hydrophobic mitochondrial proteins can be mistargeted to the endoplasmic reticulum (ER) or they can even be synthesized at the ER surface [151,152,153,154,155]. Recently, a failsafe pathway recovering mitochondrial proteins from the ER was discovered and termed ER surface-mediated protein targeting (ER-SURF) [156]. The retrieval of the ER-localized mitochondrial proteins depends on the chaperone Djp1, a cytosolic protein of the Hsp40 co-chaperone family related to the protein DnaJ of *E. coli*. Djp1 is partially localized at the ER membrane and can reroute ER-localized mitochondrial proteins to the mitochondrial outer membrane by interaction with Tom70/71. This finding is in line with another study that found that Djp1 also promotes binding of the chaperone/preprotein complex by binding to Tom70 during targeting from the cytosol [107].

## 5. Functions of Tom70 as a Mitochondrial Tether

For a long time, Tom70 was thought to act mainly or even exclusively as a receptor protein assisting the import of proteins into mitochondria. However, there is currently growing evidence that Tom70 may have a more general function as a molecular tether cooperating with some alternative molecular systems that are not related to the mitochondrial protein import machinery (Table 1).

### 5.1. Tom70 and the Endoplasmic Reticulum Protein Lam6/Ltc1

It is now well established that Tom70 and its yeast paralog Tom71 are involved in the formation of distinct membrane contact sites (MCS) between mitochondria and the endoplasmic reticulum (ER) via binding of the ER-localized membrane protein Lam6 (alternative name: Ltc1) [160,161]. Lam6 was also detected at MCS between the ER and the vacuole (the yeast lysosome) and at nucleus-vacuole junctions (NVJ). In these cases, Lam6 binds to the vacuolar protein Vac8. The mechanisms that determine the subcellular location of Lam6/Ltc1 are not known. Since the import of mitochondrial proteins is regulated by a phosphorylation site within the N-terminus of Tom70, it is conceivable that this site also plays a role in MCS formation [26]. Lam6/Ltc1 contains a GRAM/pleckstrin-homology-like (PH-like) domain, which mediates the binding to Tom70/71. With respect to the possible functions of Lam6 it is remarkable that it contains a StART domain (a steroidogenic acute regulatory protein-related lipid transfer domain) implicated in the transport of sterols [160,164,165]. Double mutants of Lam6/Ltc1 and subunits of the ER-mitochondria encounter structure (ERMES) show synthetic lethality, supporting the notion of an auxiliary function of the Tom70/71-Lam6/Ltc1 tether in lipid flux between the ER and mitochondria [160]. The ERMES complex seems to be involved in coenzyme Q_6_ synthesis and could thus be relevant for the function of the respiratory chain [166]. The ERMES complex is a structure in yeast that has no homologous counterpart in mammalian cells, but the Lam protein family is conserved both in fungi (Lam1–6) and in humans (GramD1a–c) [167].

Interestingly, the function of Tom70 as a promoter of the recruitment of ER-localized proteins to mitochondria seems to be conserved in mammals: Super-resolved STED microscopy of human cells revealed that in contrast to the import receptor TOM20, TOM70 is not distributed equally over the mitochondrial outer membrane but clusters at ER-mitochondria contact sites [58]. At the ER membrane, TOM70 interacts with the inositol 1,4,5-triphosphate receptor type 3 (IP3R3), which promotes the transfer of Ca^2+^ from the ER to mitochondria.

### 5.2. Additional Functions of Tom70

The interaction of Tom70 with the ER protein Lam6 raises the question if Tom70 may participate in additional cellular systems that are unrelated to the biogenesis of mitochondrial proteins.

New investigations indicate that this is indeed the case: In yeast, the mitochondrial surface is a site of tRNA processing catalyzed by the hetero-tetrameric tRNA splicing endonuclease (SEN) complex. Tethering of the SEN complex to mitochondria was found to be dependent on Tom70. This interaction seems to have a functional relevance as tRNA processing is reduced in Tom70 deletion strains [158,168].

Moreover, in yeast Tom70 and Tom71 are required for binding of the mitochondria-associated F-box protein Mfb1 to the mitochondrial outer surface. The molecular functions of Mfb1 are not known in detail but the protein appears to be crucial for the maintenance of mitochondrial morphology. Deletion of Mfb1 or the induction of mutations in Tom70 causes mitochondria to form aberrant aggregates of interconnected tubules [159,169,170]. The interaction of Mfb1 with Tom70 was found to be mediated by the last two C-terminal α-helices of Tom70, clearly separate from the chaperone-binding site in the Tom70 N-terminal domain [159].

**Figure 6:**
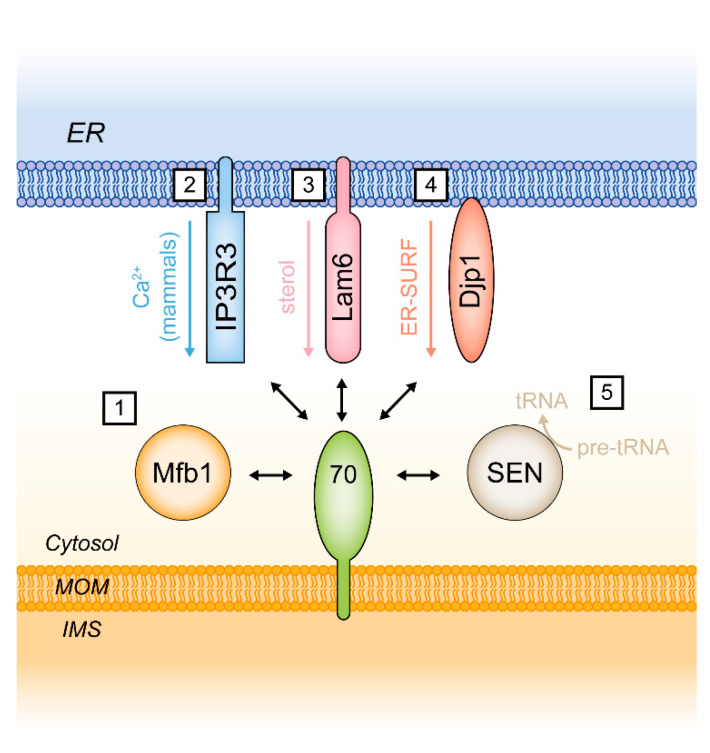
Functions of Tom70 as a molecular tether. (1) Tom70/71 recruits Mfb1 to the mitochondrial surface which is crucial for the maintenance of mitochondrial morphology. (2) TOM70 promotes ER to mitochondria Ca^2+^ transfer in association with the inositol 1,4,5-triphosphate receptor type 3 (IP3R3) in mammals. (3) In yeast, Tom70 mediates ER-mitochondria contacts via binding to sterol transporter Lam6/Ltc1. (4) In the ER surface-mediated protein targeting (ER-SURF) pathway, Tom70 facilitates the recovery of mitochondrial proteins from the ER by recognition of ER-localized co-chaperone Djp1. (5) Tom70 is required for efficient splicing of pre-tRNA at the mitochondrial surface. IMS, intermembrane space; Mfb1, mitochondria associated F-box protein 1; MOM, mitochondrial outer membrane; SEN, tRNA splicing endonuclease.

In summary, Tom70 is obviously acting in several different functional circuits that are far more diverse than anticipated only a few years ago (Figure 6). Correspondingly, human TOM70 is increasingly recognized as a relevant mitochondrial protein in different pathological contexts.

## 6. TOM70 in Health and Disease

Novel pathogenic mechanisms involving mitochondria are currently discovered across all medical disciplines, including oncology, neurology, cardiology and immunology [171,172,173,174]. Investigations on mechanisms that affect the functions of TOM70 can take advantage of the fact that TOM70 and the associated protein import machinery are highly conserved from yeast to humans [52] (Table 2).

### 6.1. A Role of TOM70 in Macrophages Infected by Leishmania Donovali

Mitochondria play a pivotal role in regulation and triggering apoptosis [179] and components of the mitochondrial protein import machinery participate in the corresponding mechanisms [180,181,182,183,184,185,186]. A specific function of TOM70 related to the regulation of cell death was observed in studies on macrophages infected by the parasite *Leishmania donovali*: The parasite promotes its survival in infected macrophages by causing an enhanced expression of MCL-1 (myeloid leukemia cell differentiation protein-1). MCL-1 targets TOM70 at the surface of mitochondria and subsequently acts as an inhibitor of apoptosis due to its structural similarity to Bcl-2. Interestingly, MCL-1 binds to TOM70 due to an internal EELD motif, which resembles the EEVD motif found at the C-terminus of cytosolic chaperones [175,187]. The prevention of host cell apoptosis by exploitation of the anti-apoptotic Bcl-2 family protein MCL-1 is also a feature of some other intracellular pathogens including *Staphylococcus aureus, Chlamydia trachomatis* and *Mycobacterium tuberculosis* [188,189,190]. Moreover, an overexpression of MCL-1 is observed in many human cancer cells and MCL-1 is therefore considered a possible target in cancer therapy [191].

### 6.2. MAVS and RIG-I: TOM70 as a Mediator of an Innate Immune Response to Viral Infections

**Figure 7:**
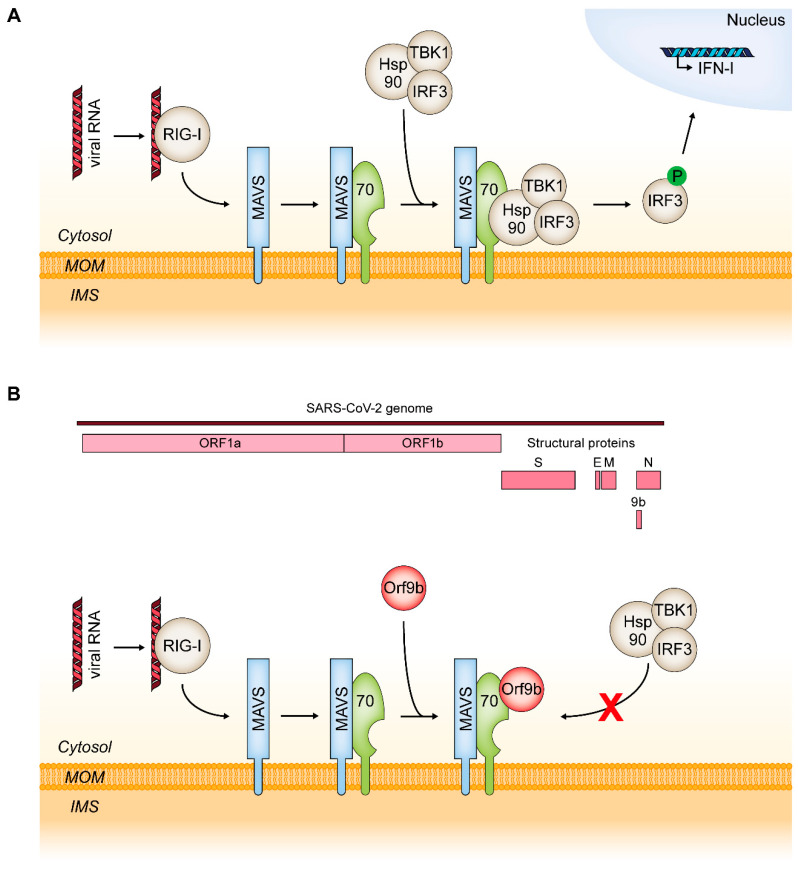
Function of TOM70 in antiviral signaling. (**A**): Upon RNA virus infection, helicase RIG-I binds to dsRNA which induces a conformational change that allows RIG-I to associate with mitochondria localized MAVS. This triggers MAVS to bind to TOM70 which subsequently leads to binding of the Hsp90/TBK1/IRF3 complex and formation of a MAVS/TOM70/Hsp90/TBK1/IRF3 supercomplex. TBK1 phosphorylates IRF3 which subsequently dissociates from the supercomplex and translocates into the nucleus to promote transcription of genes encoding proteins such as type I interferons (IFN-I). (**B**): Orf9b is an alternative reading frame on the SARS-CoV-2 RNA genome encoding for an 11 kDa protein which binds to TOM70 and inhibits the antiviral IFN-I response. IFN-I, interferon type I; IRF3, interferon regulatory factor 3; MAVS, mitochondrial antiviral signaling protein; RIG-I, encoded by the retinoic acid-inducible gene 1; TBK1, TANK-binding kinase 1.

The initiation of cell death is not only a defense mechanism against intracellular bacteria or parasites but also a common reaction to viral infections. TOM70 is a critical element in a signal cascade leading to an innate immune response specifically to viral RNA. The viral RNA is initially recognized by the cytosolic helicase RIG-I (encoded by the retinoic acid-inducible gene 1), which undergoes a conformational change and associates with MAVS (the mitochondrial antiviral signaling protein) [192,193]. This triggers MAVS to associate with TOM70 which initiates the recruitment of Hsp90, TANK-binding kinase 1 (TBK1) and interferon regulatory factor 3 (IRF3). Within this complex, the C-terminal EEVD motif of Hsp90 binds to the N-terminal TPR clamp-type domain of TOM70 [27]. IRF3 is subsequently phosphorylated by TBK1 which leads to its dissociation from the complex. The phosphorylated IRF3 translocates into the nucleus and promotes the transcription of genes encoding type I interferons (IFN-I) (Figure 7A). Moreover, binding of MAVS to TOM70 can also trigger NF-κB signaling [27] and Bax-dependent apoptosis [194].

A hallmark of SARS-CoV-2 pathogenicity is a surprisingly weak IFN-I response and it was suggested that this is a main driver of COVID-19 (coronavirus disease 2019) [195,196]. A recent report found that in SARS-CoV-2-infected cells a protein encoded by an alternative open reading frame within the nucleocapsid gene of the virus RNA, Orf9b, localizes to mitochondria and suppresses the antiviral IFN-I response by association with TOM70 [28] (Figure 7B). These results are in line with an earlier study screening for interactions of SARS-CoV-2 proteins with host cell proteins using affinity-purification mass spectrometry which likewise found Orf9b to associate with TOM70 [197]. Since the interaction of Hsp90 with TOM70 is crucial for IFN-I induction, a competition of Orf9b and Hsp90 was suggested by Jiang et al. (2020) as a possible inhibition mechanism. However, in their study they found that Orf9b also associates with a TOM70 variant lacking the N-terminal clamp-type TPR domain necessary for chaperone binding. The molecular consequences of Orf9b binding to TOM70 therefore remain to be established.

Given the function of Tom70/71 in the formation of MCS with the ER by docking to Lam6/Ltc1 in yeast and IP3R3 in mammals, it is tempting to speculate about a role of membrane contacts in the pathogenicity of SARS-CoV-2. The TOM70-IP3R3 interaction is crucial for Ca^2+^ transfer from the ER to mitochondria and mitochondrial Ca^2+^ overload is widely regarded as a trigger of apoptosis [58,198]. Moreover, the data of Filadi et al. (2018) suggest that impaired Ca^2+^ transfer to mitochondria induces macroautophagy, which was found to act as a pro survival mechanism in an earlier study [199]. In fact, it has already been reported that viral proteins can indeed be targeted to and restructure ER-mitochondria contact sites [200]. It is conceivable that Orf9b does not only compete with proteins of the IFN-I inducing cascade, but also with ER-localized proteins to disrupt ER-mitochondria MCS and thus inhibits induction of cell death.

### 6.3. TOM70 in the Regulation of Mitochondrial Functions and ROS Formation in Heart Cells

The recent years have seen several studies linking TOM70 to the mechanisms of recovery after myocardial infarction and to some cardiomyopathies:

A screen of mRNA transcription and protein levels revealed a downregulation of TOM70 expression in human pathological hypertrophic cardiac samples and in rat model systems for cardiac hypertrophy [176]. The authors of this study were able to induce cardiomyocyte hypertrophy by knockdown of TOM70 and to reduce it by overexpression of TOM70, both in vitro and in vivo. TOM70 knockdown caused high levels of reactive oxygen species (ROS) and diminished import of an important mediator of mitochondrial fusion, the inner membrane protein Opa1 (optical atrophy-1), which resulted in an abnormal mitochondrial morphology.

These findings are in line with a report on a TOM70 deficiency causing mitochondrial damage and ROS overload and thereby aggravated injury after myocardial infarction (post-MI) in mice hearts [177,201]. The authors found that administration of antioxidant N-acetylcysteine (NAC) or melatonin reversed the effects of a TOM70 knockdown. The latter correlated with an upregulation of TOM70 expression, again confirming an involvement of TOM70 in the regulation of mitochondrial ROS production.

Consistently, both mRNA levels and protein expression of TOM70 were reported to be reduced in heart cells of diabetic mice [202]. In line with the results from Li et al. (2014) and Pei et al. (2017), knockdown of TOM70 led to exacerbated cardiac hypertrophy and fibrosis in this animal model as well as increased high-glucose high-fat (HGHF)-induced ROS levels and apoptosis in neonatal cardiomyocytes.

These studies did not reveal the molecular mechanisms that determined the relevance of TOM70 in the cellular functions, but they demonstrate that the presence or absence of TOM70 can be a limiting factor in cardiac functions and pathologies. The expression of TOM70 in heart myocytes seems to be generally reduced in older individuals [203]. In this regard, it would be interesting to further investigate the possible impact of post-translational modifications in TOM70, since TOM70 was reported to be phosphorylated at serine 94 in a rat model of heart failure [204]. It is known from studies with yeast cells that the phosphorylation of a single serine side chain can regulate the activity of Tom70 [26].

### 6.4. Functions of TOM70 in Mitochondrial Quality Control

Mitochondria are not only a source of ATP but also of ROS and they can easily be damaged by the ROS that is produced by their own respiratory chain [205]. Aberrant mitochondria are removed by a selective form of autophagy, termed mitophagy, which is a crucial mechanism in mitochondrial quality control [206]. Dysfunctional mitochondria and impaired mitophagy are observed in many pathological conditions and the mechanisms and implications of mitophagy were investigated in molecular detail particularly with respect to Parkinson’s disease (PD), amyotrophic lateral sclerosis (ALS) and Alzheimer’s disease (AD) [207,208,209].

In humans and in other metazoans, certain types of stress-induced mitophagy are regulated by the interplay of two key proteins, the mitochondrially localized kinase PINK1 (PTEN-induced putative kinase 1) and the cytosolic E3 ubiquitin ligase Parkin [210]. Mutations in their genes, *PINK1* and *PARK2* respectively, are associated with forms of early-onset Parkinson’s disease. The PINK1/Parkin system acts as a sensor of mitochondrial quality and is activated particularly after loss of the mitochondrial membrane potential [211].

Under normal conditions, the mitochondrial presequence-containing protein PINK1 is imported into mitochondria via the TOM and TIM23 complexes and then cleaved by the matrix processing peptidase (MPP) and a protease of the rhomboid type in the inner membrane (the PINK1/PGAM5 associated rhomboid-like protease, PARL). The resulting cleavage product is then released into the cytoplasm and degraded by the proteasome. Upon mitochondrial damage, protein import is compromised and PINK1 accumulates at the mitochondrial outer membrane in a complex including TOM70, TOM20, TOM22, and TOM40. Stalling at the outer membrane causes PINK1 to phosphorylate Parkin which increases its E3 ligase activity and ultimately leads to the initiation of mitophagy [212]. TOM70 is described as the main import receptor for PINK1 and consistently an enhanced mitophagic flux is observed after deletion of *TOMM70* [162,178]. Moreover, TOM70 was identified as an interaction partner of Parkin and to be ubiquitinylated across all its TPR motifs in a Parkin-dependent fashion [213,214]. The ubiquitinylated TOM70 is suggested to participate in the recruitment of proteins crucial for the formation of the autophagosome [178,215].

Eventually, it was found that not only complete mitochondria but also mitochondria-derived vesicles (MDVs) can be transported to lysosomes for subsequent degradation, and Tom70 was already shown to play a role in this pathway in yeast [216,217,218].

### 6.5. Mitochondriopathies Caused by Mutations in TOMM70

Mutations in nuclear or mitochondrial genes encoding for mitochondrial proteins can lead to different mitochondria-associated diseases (mitochondriopathies) with a chronic, progressive course and a varying onset between birth and late adulthood [2,219]. The recently discovered roles of TOM70 in many pathological contexts suggest that also mutations in the *TOMM70* gene could be involved in the development of such mitochondriopathies in humans. Indeed, two recent studies reported on mutations in *TOMM70* that caused neurological impairment, developmental delay, severe anemia, and lactic acidosis [172,220]. The clinical consequences of these mutations indicate that essential mitochondrial functions were substantially impaired.

## 7. Conclusions

The first reports on a functional characterization of the mitochondrial outer membrane protein Tom70 were published in 1990 [12,14], in a time when no genome of any organism had been sequenced and proteome analysis was not yet possible. Under these conditions, Tom70 was characterized as a protein of isolated mitochondria and only in respect to a single function. It was concluded that Tom70 is a protein with the specific function to direct the members of a limited class of newly synthesized preproteins to the general import pore of the mitochondria, thereby serving a limited but well defined and specific purpose in the cell. Tom70 was known as a protein in two different fungi, yeast and *Neurospora crassa*, and it was unknown if human cells may have a similar protein, or possibly a completely different system of mitochondrial protein import. A few decades later, both the possibilities and the results of research projects have changed. Genome projects revealed that humans are closer related to fungi than ever anticipated, and in fact, humans definitely have a Tom70 and a protein import machinery similar to fungi. But the description of Tom70 has become more complicated. Not only is it possible that preproteins bypass Tom70 on their way to the general import channel. Some preproteins bind to Tom70 and are subsequently transferred to the outer membrane MIM complex and thus target an alternative import channel. Preproteins can interact with Tom70 although they contain a positively charged presequence, thereby belonging to a class of proteins that was originally thought to bind exclusively to Tom20. The mechanisms that determine the selectivity of Tom70 have become obscure, and it may well be that most preproteins bind to Tom70 only indirectly, mediated by the preprotein-associated chaperone proteins.

Eventually, the initial experiments with isolated mitochondria may have missed the most important functions of Tom70: It turned out that in yeast, Tom70 can act as a ligand of the endoplasmic reticulum protein Lam6, possibly in a context of lipid transfer between membranes. Eventually, TOM70 was found to provide the essential receptor structure for the mitochondrial antiviral-signaling protein MAVS, a major sensor of viral infections in the innate immune system. TOM70, probably due to this function, seems to be an appropriate target for the protein encoded by Orf9b in the genome of SARS-CoV-2, the agent of the 2020 virus pandemic. The functions of Tom70 have become confusing, but also more interesting.

## Figures and Tables

**Table 1:** Known substrates of Tom70. Listed are examples of proteins that were found to associate with Tom70 either directly or indirectly (e.g., through chaperones).

Receptor	Cellular Context	Substrate Class	Example	Reference	Subcellular Location	Function
*S. c.* Tom70	Protein import	Mitochondrial carrier family (MCF)	AAC	[92,112]	MOM, MIM	Translocation of metabolites across membranes
		β-Barrel proteins	Porin	[109]	MOM	Metabolite translocation across the MOM, VDAC in humans
		Single-membrane-spanning proteins	Atg32	[95]	MOM	Essential receptor for mitophagy in yeast
		Multi-membrane-spanning proteins	Ugo1	[96,97]	MOM	Required for mitochondrial fusion
			MPC	[94]	MIM	Translocation of pyruvate across the MIM
		Presequence-containing proteins	Aco1	[134]	Matrix	Isomerization of citrate to isocitrate via *cis*-aconitate in the TCA cycle
		iMTS proteins	Atp1, Atp25	[134,137]	MIM	Subunits of the F_1_F_O_-ATPase, contain internal mitochondrial targeting signals (iMTS)
			Ubx2	[138]	ER, MOM	Recruitment of Cdc48 for removal of arrested proteins from the TOM channel
		TIM complex subunits	Tim54	[157]	MIM	Recruitment of small Tim proteins
	Cooperation with cytosolic proteins and nucleic acids	Chaperones	Hsp70	[25]	Cytosol	Mediates association of hydrophobic preproteins with TPR receptors
		Co-chaperones	Djp1	[107]	ER, Cytosol	Co-chaperone of the Hsp40 family
		Other cytosolic factors	SEN subunits	[158]	Cytosol	Mediates tRNA splicing at the MOM
			Mfb1	[159]	Cytosol	Recruitment of Mfb1 by Tom70/71 is crucial for mitochondrial morphogenesis
			mRNA	[149]	Cytosol	Co-translational protein import
	Cooperation with other membranes	ER proteins	Lam6/ Ltc1	[160,161]	ER	Formation of ER-mitochondria contact sites via binding to Tom70/71, involved in sterol transfer
			Djp1	[156]	ER, Cytosol	ER surface retrieval pathway (ER-SURF)
*H. s.* TOM70	Protein import	Solute carrier family (SLC25)	ANT1	[111]	MIM	Mammalian homolog of the AAC
		Multi-membrane- spanning proteins	PBR	[131]	MOM	Cholesterol import into the MIM
		Presequence-containing proteins	PINK1	[162]	MOM, Matrix	Induction of mitophagy after mitochondrial depolarization in mammals
	Cooperation with cytosolic proteins and nucleic acids	Chaperones	Hsp70, Hsp90	[25]	Cytosol	Mediate association of hydrophobic preproteins with N-terminal TPR domain of TOM70
		Co-chaperones	Hsp40 family	[111]		Enhance binding of chaperones to TOM70
		Autophagy machinery	Atg2	[163]	Cytosol	Crucial for autophagosome formation
		Other cytosolic factors	mRNA	[148]	Cytosol	Co-translational protein import
	Cooperation with other membranes	ER proteins	IP3R3	[58]	ER	Ca^2+^ transfer via ER-mitochondria contact sites formed by IP3R3 and TOM70
	Signaling	Antiviral signaling	MAVS	[27]	MOM	Involved in antiviral signaling cascade triggering an innate immune response
		Viral proteins	Orf9b	[28]	MOM	Alternative ORF of nucleocapsid (N) gene of SARS-CoV-2, suppresses IFN-I response via binding to TOM70

AAC, ADP/ATP carrier; Aco1, aconitate hydratase; ANT1, adenine nucleotide transporter 1; Djp1, DnaJ-like protein 1; ER, endoplasmic reticulum; Hsp, heat shock protein; IFN-I, interferon type I; IP3R3, inositol 1,4,5-triphosphate receptor type 3; Ltc1, Lipid transfer at contact site protein 1; MAVS, mitochondrial antiviral-signaling protein; Mfb1, mitochondria associated F-box protein 1; MOM, mitochondrial outer membrane; MIM, mitochondrial inner membrane; MPC, mitochondrial pyruvate carrier; PBR, peripheral benzodiazepine receptor; PINK1, Phosphatase and tensin homolog-induced kinase 1; SEN, tRNA splicing endonuclease; Ubx2, Ubx domain-containing protein 2; VMC1, viral mitochondrial carrier 1.

**Table 2:** Implications of TOM70 in human health and disease.

Implication	Associated Disease	Involvement of TOM70	Reference
*Leishmania donovani* infection	Leishmaniosis	Suppression of apoptosis by mediating import of anti-apoptotic protein MCL-1	[175]
SARS-CoV-2 infection	COVID-19	Suppression of IFN-I inducing antiviral RIG-I/MAVS cascade through inhibition of TOM70 by Orf9b	[28]
Cell survival	Cancer	Ca^2+^ transfer from ER to mitochondria by binding to IP3R3	[58]
Pathological hypertrophy	Heart failure	Downregulation of *TOMM70* leads to increased ROS levels and diminished Opa1 import	[176]
Post-MI injury	Heart failure	TOM70 is essential for melatonin-induced protection against post-MI injury	[177]
Mitochondrial quality control	PD, ALS, AD	TOM70 is involved in PINK1 import and formation of the TOM/PINK1/Parkin complex upon mitochondrial depolarization	[162,178]

AD, Alzheimer’s disease; ALS, amyotrophic lateral sclerosis; COVID-19, coronavirus disease 2019; IFN-I, interferon type I; IP3R3, inositol 1,4,5-triphosphate receptor type 3; MAVS, mitochondrial antiviral signaling protein; MCL-1, myeloid leukemia cell differentiation protein-1; MI, myocardial infarction; Opa1, optic atrophy 1; PD, Parkinson’s disease; PINK1, Phosphatase and tensin homolog-induced kinase 1; RIG-I, protein encoded by the retinoic acid-inducible gene 1; ROS, reactive oxygen species; SARS-CoV-2, severe acute respiratory syndrome coronavirus 2.

## Abbreviations

AAC	ADP/ATP carrier
ADP	Adenosine diphosphate
ATP	Adenosine triphosphate
BN-PAGE	Blue native-polyacrylamide gel electrophoresis
ER	Endoplasmic reticulum
Hsp	Heat shock protein
IFN-I	Interferon type I
IMS	Intermembrane space
IP3R3	Inositol 1,3,4-triphosphate receptor type 3
Lam6	Lipid transfer protein anchored at a membrane contact site
Ltc1	Lipid transfer at contact site 1
MAVS	Mitochondrial antiviral signaling protein
MCF	Mitochondrial carrier family
MCL-1	Myeloid cell Leukemia 1
Mfb1	Mitochondria-associated F-box protein 1
MIM	Mitochondrial inner membrane
MIM complex	Mitochondrial import complex
MOM	Mitochondrial outer membrane
RIG-I	Retinoic acid-inducible gene 1
ROS	Reactive oxygen species
SARS-CoV-2	Severe acute respiratory syndrome coronavirus 2
SEN	tRNA splicing endonuclease
SLC25	Solute carrier family 25
TIM	Translocase of the inner mitochondrial membrane
TOM	Translocase of the outer mitochondrial membrane
TOM70	Mammalian homolog of yeast Tom70
